# Postmenopausal Deep Infiltrating Endometriosis of the Colon: Rare Location and Novel Medical Therapy

**DOI:** 10.1155/2018/9587536

**Published:** 2018-02-14

**Authors:** Benjamin M. Snyder, Joseph W. Beets, Bruce A. Lessey, Samuel R. W. Horton, Gary A. Abrams

**Affiliations:** ^1^University of South Carolina School of Medicine Greenville, Greenville, SC, USA; ^2^Department of Gastroenterology/Liver Center, Greenville Health System, University of South Carolina School of Medicine Greenville, Greenville, SC, USA; ^3^Department of Obstetrics and Gynecology, Greenville Health System, University of South Carolina School of Medicine Greenville, Greenville, SC, USA; ^4^Department of Pathology, Greenville Health System, University of South Carolina School of Medicine Greenville, Greenville, SC, USA

## Abstract

We report an uncommon case of deep infiltrating endometriosis of the colon presenting as iron deficiency anemia nine years after hysterectomy with bilateral salpingo-oophorectomy. The endometrial implant was found at the hepatic flexure, an exceedingly rare location for endometriosis invasion with no cases distinctly reported in the literature. Additionally, the presentation of gastrointestinal endometriosis as iron deficiency anemia is not well documented in the literature. Instead of surgery, we prescribed a novel medical therapeutic approach using conjugated estrogen-bazedoxifene to antagonize the proliferative effects of estrogen on endometrial tissue. After five months of therapy and repeat colonoscopy, no evidence of endometrial tissue remained in the hepatic flexure.

## 1. Introduction

Endometriosis is a common disorder in women of reproductive age occurring in 8–10% [1]. However, endometriosis has not been significantly studied in postmenopausal women. The development of endometriosis after menopause is a rare phenomenon, and it has often been reported in the setting of hormone replacement therapy (HRT) [2] and treatment with tamoxifen [3, 4]. While postmenopausal endometriosis is uncommon, deep infiltrating endometriosis involving the colon in postmenopausal women has been noted only in the case report literature [5–9]. We describe a case of a postmenopausal woman presenting with iron deficiency anemia due to colonic endometrial infiltration and a novel therapeutic approach.

## 2. Case Report

**Figure 1:**
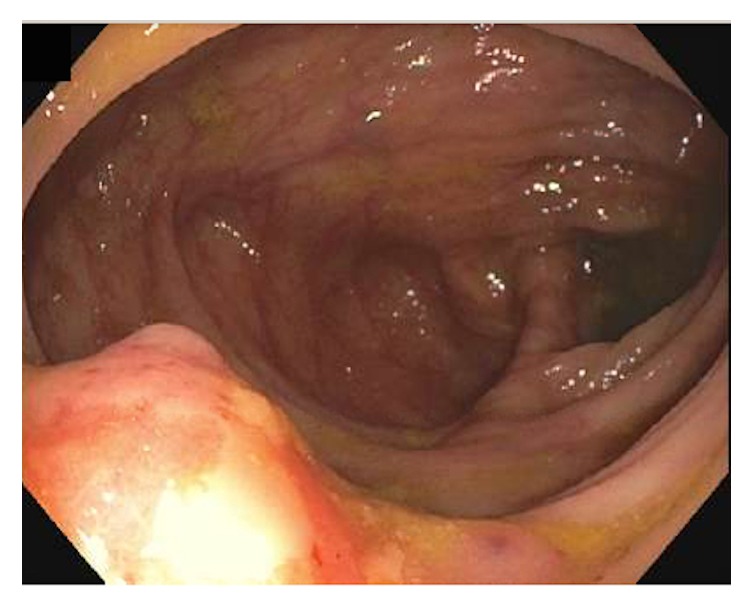
The nodule was visualized at the hepatic flexure with mild ulceration and bleeding at the surface. Ulceration and blood loss were the presumed explanation for the patient's chronic iron deficiency.

**Figure 2:**
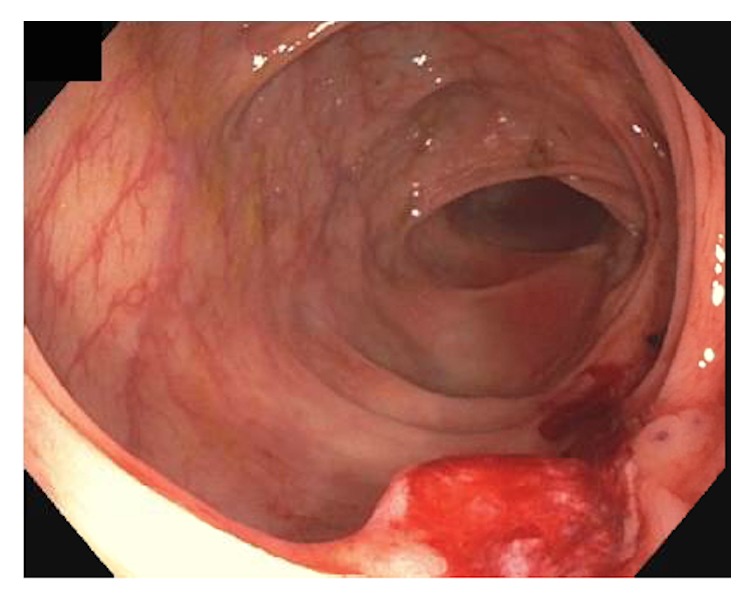
The nodule was partially removed by cold snare polypectomy but was not amenable to complete removal under endoscopy.

A 60-year-old Caucasian woman was referred to gastroenterology department due to three years of persistent iron deficiency anemia (IDA) resistant to oral iron supplementation with patient-reported blood in the stool. Her history was significant for endometriosis in early reproductive years and uterine fibroids treated by hysterectomy with bilateral salpingo-oophorectomy nine years before her current presentation. She was given oral estradiol therapy as part of hormone replacement therapy (HRT). She underwent an esophagogastroduodenoscopy (EGD) that was of normal result and a colonoscopy that revealed moderately severe diverticulosis and a nodular ulcerated lesion at the hepatic flexure (Figure 1) that was partially removed by hot snare cauterization (Figure 2). This was not a complete resection and allowed for better tissue sampling. The nodule appeared deeper than what could be visualized by endoscopy and was biopsied multiple times. India ink was used to tattoo the location of the nodule for a follow-up examination.

**Figure 3:**
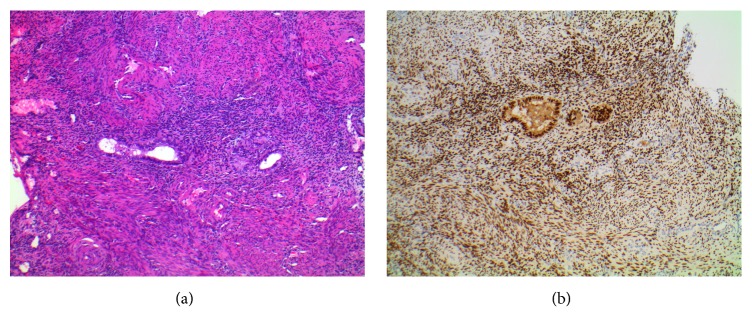
(a) Staining of the colonic nodule shows active proliferative endometrial glands and stromal tissue (hematoxylin and eosin, 40x). (b) Immunohistochemistry demonstrates diffusely positive staining for the estrogen receptor in a pattern consistent with endometrial tissue (100x).

Histological analysis of the biopsies noted mural endometriosis and adenomatous tissue within the endometrial tissue and surrounding mucosa (Figure 3). The endometrial tissue infiltrated the full thickness of the biopsy specimen, which extended through the submucosa. The presence of endometrial tissue through the depth of the biopsy, extending into the mucosa, is indicative of complete thickness penetration of the colonic wall. Though the patient experienced no other symptoms of endometriosis, deep infiltrating endometriosis was a likely explanation for her IDA. Gynecology was consulted, and the patient's estradiol therapy was discontinued. Given that the subject had few physical symptoms of her disease but had previously suffered vasomotor symptoms of menopause, medical treatment was an appropriate approach for her condition. A course of conjugated estrogen-bazedoxifene (CE/BZA) (Duavee: 0.45–20 mg, Pfizer, New York, NY) was selected instead of surgery. CE/BZA is commonly used in women with a uterus who desire HRT, as it prevents estrogen from stimulating endometrial tissues using the selective estrogen receptor modulator, bazedoxifene.

**Figure 4:**
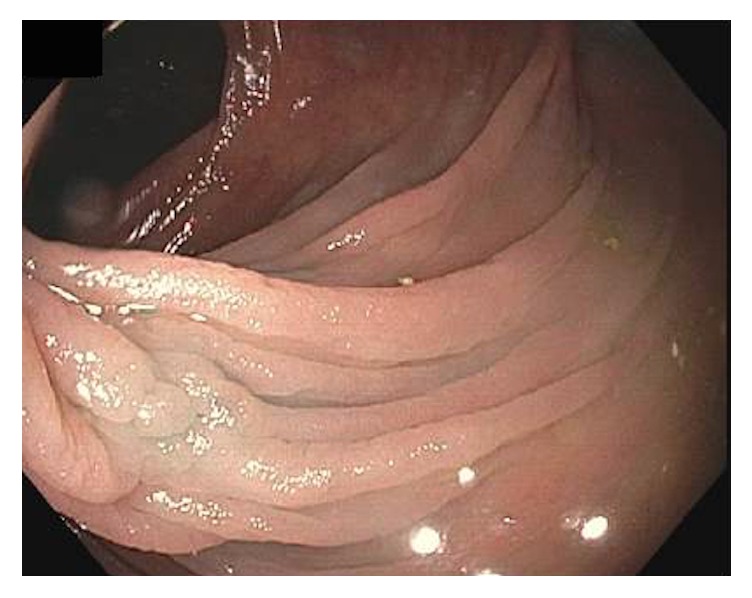
Approximately eight months after cold snare polypectomy and treatment with conjugated estrogen/bazedoxifene, no evidence of the nodule remained at the marked site at the hepatic flexure of the colon.

Approximately three months after her original visit to gastroenterology department and consultation with hematology, the patient received two intravenous iron infusions one week apart in conjunction with the course of CE/BZA therapy. Follow-up colonoscopy, eight months after the initial colonoscopy and five months of CE/BZA therapy, demonstrated scarring from the initial procedure and no residual nodule at the hepatic flexure (Figure 4). Biopsies taken identified fragments of tubular adenoma without any evidence of remaining endometriosis suggesting complete regression. Follow-up labs revealed normalization of iron studies, anemia, and microcytosis. Iron infusion and complete regression of endometrial tissue and reversal of minor blood loss from associated ulceration are the presumptive explanation for resolution of this patient's iron deficiency anemia.

## 3. Discussion

Deep endometriosis (also called deep infiltrating endometriosis) is a form of endometriosis that invades any vital structures such as bowel, ureters, or bladder [10]. Intestinal involvement of endometriosis has been estimated to exist in between 3.8% and 37% of patients with an endometriosis diagnosis [11]. The most common sites of bowel endometriosis are rectum, ileum, appendix, and cecum from most to least prevalent, while some cases of gastric and transverse colonic involvement have been reported [11]. Endometriosis is often difficult to diagnose due to the generalized nature of symptoms: pelvic pain, dysmenorrhea, dyspareunia, and dysuria/dyschezia [12]. Symptoms associated with bowel endometriosis are abdominal pain relieved with defection, change in frequency or appearance of stool, and abnormal rectal bleeding, but most of these symptoms are seen only in cases of mucosal involvement [13]. Additionally, it is difficult to diagnose bowel endometriosis even with colonoscopy as most cases do not infiltrate beyond the serosa and very few infiltrate the mucosa [14]. With a wide range of presentations and the often asymptomatic disease course, until it significantly progressed, deep infiltrating endometriosis is a complicated disease to diagnose and treat.

The treatment of endometriosis has primarily focused on alleviating symptoms and restoring fertility in women of reproductive age [15–17], but to this point, no curative therapy exists. To date, research has primarily focused on the surgical treatment of deep endometriosis with fewer resources being devoted to understanding the medical management of the disease. Specifically, research in postmenopausal endometriosis has purported to indicate the necessity of resection due to the risk of malignancy [18, 19]. However, recurrence of the disease is possible even with surgical intervention, and there is a risk of significant complications as a result of surgical resection especially in bowel endometriosis [20]. There has been a rise in invasive bowel resection for treatment of colorectal endometriosis in recent years, and some authors have begun to question the efficacy of such a practice in favor of less invasive interventions such as ablation or medical management [21–23]. Complete excision of endometriosis of the bowel, more than ablation, has been shown to reduce some symptoms such as dyspareunia, but overall symptoms are decreased nearly identically with ablation or excision [24]. The difficulty of completely eliminating endometriosis of the bowel and permanently reducing symptoms is the preferential association of endometriosis tissue with the enteric nervous system, which also accounts for the set of symptoms seen in deep infiltrating endometriosis of the bowel [13, 25, 26]. Additionally, macronodules of endometriosis may be readily visible during surgery, but micronodules are identified in histology making complete excision of endometriosis unlikely [27]. As discussed here, many complicating factors are associated with surgical intervention in deep infiltrating endometriosis in a postmenopausal population. A more patient-oriented approach evaluates specific patient factors as in this case, demonstrating a less invasive method, that is, ablation followed by CE/BZA therapy in this case.

Medical management of endometriosis and especially postmenopausal endometriosis is evolving. The currently postulated mechanisms of premenopausal endometriosis include retrograde menstruation, coelomic metaplasia, immune deficiencies, and Mullerian remnants, but these do not adequately account for all cases in women of reproductive age [17]. Postmenopausal endometriosis cannot be fully explained by the postulated mechanisms, but high levels of ectopic estrogen production from nonovarian tissue can activate endometrial tissue [28]. Aromatase has been shown to have a role in the pathogenesis of endometriosis and has long been a proposed target for treatment [29]. Few cases of the treatment of postmenopausal endometriosis with aromatase inhibitors (AI) have been reported; however they suggest that significant symptom reduction is possible with AIs [18]. One case even notes the positive effects of AIs on abdominal endometriosis and suggests a potential role for AIs in the treatment of postmenopausal deep infiltrating endometriosis [30]. Although there are articles demonstrating the usefulness of AIs in postmenopausal endometriosis, empiric research is lacking.

CE/BZA was approved in 2013 for treatment of the vasomotor symptoms associated with menopause as well as the treatment of postmenopausal osteoporosis by a non-hormone replacement therapy/menopausal hormone therapy mechanism [31]. CE/BZA is a combination of conjugated estrogen and bazedoxifene, a selective estrogen receptor modulator (SERM) that induces degradation of estrogen receptors in breast and endometrial tissue [32]. Original clinical trials validated the efficacy of CE/BZA in reduction of menopausal vasomotor symptoms and protection against osteoporosis, while demonstrating the protective effects of bazedoxifene on breast and endometrial tissue in the presence of estrogen [33]. Research has also shown that CE/BZA can cause regression of endometriosis in a murine model [34, 35]. It was by this mechanism hypothesized that CE/BZA would be an ideal option for medical management in this postmenopausal patient thereby inducing regression of endometrial tissue while providing the protective bone effects of estrogen and preventing vasomotor symptoms of menopause.

In conclusion, this case highlights an unusual presentation of iron deficiency anemia due to a rare colonic location of deep infiltrating endometriosis in a postmenopausal woman successfully treated medically with a novel and less invasive approach (CE/BZA) that provides mechanisms for both symptom control and disease regression.
